# Psychological stress factors and salivary cortisol in nursing students throughout their training^*^

**DOI:** 10.1590/1980-220X-REEUSP-2022-0078en

**Published:** 2022-12-16

**Authors:** Sandra Soares Mendes, Milva Maria Figueiredo De Martino, Filipy Borghi, Camila Maiara Rocha-Teles, Aglecio Luiz de Souza, Dora Maria Grassi-Kassisse

**Affiliations:** 1 Universidade Estadual de Campinas, Faculdade de Enfermagem, Programa de Pós-Graduação em Enfermagem, Campinas, SP, Brazil.; 2 Universidade Estadual de Campinas, Instituto de Biologia, Laboratório de Estudos do Estresse. Campinas, SP, Brazil.; 3 Universidade Estadual de Campinas, Faculdade de Ciências Médicas, Departamento de Clínica Médica, Campinas, SP, Brazil.

**Keywords:** Stres, Psychologica, Hydrocortison, Saliv, Student, Nursing, Estrés Psicológico, Hidrocortisona, Saliva, Estudiantes de Enfermería, Estresse Psicológico, Hidrocortisona, Saliva, Estudantes de Enfermagem

## Abstract

**Objective:**

to analyze psychological stress factors and salivary cortisol concentration in nursing undergraduates throughout their training.

**Method:**

a cross-sectional, analytical, and comparative study carried out in an evening course using a sociodemographic questionnaire, an Instrument to Assess Stress in Nursing Students, and salivary cortisol analysis. The study included descriptive and comparative analyses and a multiple linear regression model.

**Results:**

187 participants answered the questionnaires, and 129 had their cortisol quantified. The domains Practical Activities Execution, Professional Communication, and Professional Training represented the stress factors with the highest mean values for 3^rd^, 4^th^, and 5^th^-year students compared to 1^st^ and 2^nd^ year. For the 5^th^ year, it was the domains Professional Communication and Professional Training compared to the 3^rd^ year and Environment compared to the 1st and 3rd year. A significant result was obtained between the times of cortisol collections for males (p < 0.0001), females (p < 0.0001), and for 1^st^ (p = 0.0319) 2^nd^ (p = 0.0245), and 5^th^ (p < 0.0001) years.

**Conclusion:**

Students in years 3 through 5 had higher exposure to stressors, and there were adjustments in cortisol production rhythmicity for students in years 1, 2, and 5.

## RESUMO

**Objetivo:**

analisar os fatores de estresse psicológico e a concentração de cortisol salivar de graduandos de Enfermagem ao longo da formação.

**Método:**

estudo transversal, analítico e comparativo realizado em curso noturno por meio de questionário sociodemográfico, Instrumento para Avaliação de Estresse em Estudantes de Enfermagem e análise do cortisol salivar. Foram feitas análises descritivas, comparativas e modelo de regressão linear múltipla.

**Resultados:**

um total de 187 responderam aos questionários, e 129 tiveram o cortisol quantificado. Os domínios Realização das Atividades Práticas, Comunicação Profissional e Formação Profissional representaram os fatores de estresse com os maiores valores médios para alunos do 3º, 4º e 5º anos em comparação ao 1º e 2º anos. Para o 5º ano, foram os domínios Comunicação Profissional e Formação Profissional em relação ao 3º ano e Ambiente em comparação ao 1º e 3º anos. Obteve-se resultado significativo entre os horários das coletas de cortisol para homens (p < 0,0001), mulheres (p < 0,0001) e para o 1º (p = 0,0319) 2º (p = 0,0245) e 5º (p < 0,0001) anos.

**Conclusão:**

alunos do 3º ao 5º ano tiveram maior exposição aos fatores de estresse, e houve ajustes na ritmicidade de produção do cortisol para alunos do 1º, 2º e 5º anos.

## RESUMEN

**Objetivo:**

analizar los factores de estrés psicológico y la concentración de cortisol salivar de estudiantes de enfermería a lo largo de su formación.

**Método:**

es un estudio transversal, analítico y comparativo realizado en un curso nocturno mediante cuestionario sociodemográfico, Instrumento para Evaluación del Estrés en Estudiantes de Enfermería y análisis del cortisol salivar. Se llevaron a cabo diversos análisis descriptivos, comparativos y modelo de regresión linear múltiple.

**Resultados:**

187 respondieron a los cuestionarios y a 129 se les cuantificó el cortisol. Los dominios Realización de Actividades Prácticas, Comunicación Profesional y Formación Profesional representaron los factores de estrés con los valores medios más altos en los estudiantes de 3^er^, 4º y 5º año en comparación con los de 1^er^ y 2º año. Para el 5º año fueron los dominios Comunicación Profesional y Formación Profesional en comparación con el 3^er^ año y Medio Ambiente en comparación con el 1^er^ y 3^er^ año. Se obtuvo un resultado significativo entre los momentos de las recogidas de cortisol de los hombres (p < 0,0001), de las mujeres (p < 0,0001), y del 1^er^ (p = 0,0319), 2º (p = 0,0245) y 5º (p < 0,0001) año.

**Conclusión:**

los alumnos de 3^er^ a 5º año tuvieron una mayor exposición a los estresores y hubo ajustes en la ritmicidad de la producción de cortisol en los alumnos de 1^er^, 2º y 5º año.

## INTRODUCTION

The global scenario shows that undergraduate nursing students experience high levels of stress with harmful repercussions for their health, curricular activities, and emotional state^(1)^. High levels of stress and impairment in quality of life were identified in nursing students in the Philippines, Greece, and Nigeria^(2)^, and in Brazil, the overall stress level analysis of nursing students from a public institution revealed that 58.7% presented medium/high level of stress^(3)^.

Stress is a non-permanent relationship between the individual and their environment, in which the subject evaluates the stressful event or agent as a threat that goes beyond their efforts and adaptive coping resources^(4)^. Importantly, stress does not always pose a threat to a person’s health and well-being, stress can help in coping with challenges^(5)^. However, chronic stressful clinical conditions can make the body more susceptible to various health conditions, such as hypertension, diabetes, and depression^(6)^.

Variability in human physiological responses to stress occurs through activation of the Sympathetic Nervous System and the Hypothalamus-Pituitary-Adrenal (HPA) neuroendocrine axis, which plays a key role in the response to external and internal stressors by regulating the cortisol level. The stressor agent promotes the HPA axis activation, which increases cortisol^(7)^. Cortisol production follows a 24-hour circadian rhythm, characterized by high levels upon awakening and a subsequent increase in the first 30 minutes, with gradual reduction throughout the day^(8)^, both in the blood, urine, and saliva^(7)^. Thus, cortisol is usually used as a biomarker of psychological stress as well as associated mental or physical alterations^(9)^. International research has demonstrated the quantification of salivary cortisol concentration in the stress evaluation in this population^(10,11)^.

In the nursing training environment, some conditions can be potentially more stressful, such as the student’s initial contact with the university^(12)^, curricular and extracurricular demands and assessments, the relationship with teachers, the conflicting interaction with colleagues and health professionals^(13)^, the situations experienced in the internship fields inherent to care, which can generate reactions of rejection, anxiety, and emotional imbalance such as mourning and death^(14)^, the problems related to public transportation in the commute between housing, internship fields, and college, among others^(15)^. In addition, the need to balance work and study is a reality experienced by many students and identified in courses offered at night or in a single day period, and this condition is a contributing factor to stress manifestation^(16)^.

Assessing psychological stress in nursing students has been performed by different psychometric instruments, in other words, questionnaires that indicate stress and its impact during the academic training period^(3,17)^. However, the response to stress from a physiological perspective through the dosage of salivary cortisol in this population in the Brazilian scenario is scarce. Thus, this study aimed to analyze the psychological stress factors and the concentration of salivary cortisol in undergraduate nursing students during their training.

## METHODS

### Study Design

This is a cross-sectional, analytical, comparative study.

### Study Site

The study was carried out in a private institution of higher education, located in Poços de Caldas, Minas Gerais state.

### Population

The population included students of a Nursing course offered in the evening. We included students over 18 years old regularly enrolled from the 1^st^ to the 5^th^ year. Exclusion criteria were students taking corticoids or any medication that could interfere with the increase or decrease in cortisol concentrations, such as anti-inflammatory drugs, as well as students on medical or maternity leave. The sample calculation was conducted, considering a proportion equal to 0.5, a 5% sampling error, a 5% significance level, and a population size of 192 students. The calculation resulted in a sample of 141 students. A calculation was performed considering the methodology for estimating sample size for a multiple linear regression model. In this calculation 5 independent variables were considered, a significance level of 5%, a test power of 80%, and an effect size equal to 0.15, which can be considered an effect size of medium degree. The calculation resulted in a sample of 92 students. Among the 192 students, 4 refused to participate, and the others were not present or were on medical or maternity leave. The final sample consisted of 187 students who responded appropriately to the questionnaires. However, 23 students did not deliver saliva samples, 14 did not follow the established collection protocol, 20 samples were excluded after centrifugation due to insufficient saliva for analysis (volume less than 5uL), and 1 sample was discarded because it had a very altered reading, which was considered an “outlier”, preventing statistical analysis. Therefore, the final salivary cortisol sample was composed of 129 students.

### Data Collection

Data collection occurred during the first semester of 2018, from April to June, with prior authorization from the course coordination, which established with the teachers the days and times for data collection. The students were approached in the classroom at night, or in meeting rooms at the internship field in the morning and afternoon, in order to formalize the invitations to students, clarify doubts, as well as detail collection procedures and inclusion and exclusion criteria, and the topic on the use of corticosteroids and anti-inflammatory drugs was also addressed at this moment.

The students answered a self-reported questionnaire to obtain sociodemographic data, and to evaluate psychological stress the Instrument for Assessment of Stress in Nursing Students (ASNS[*AEEE*]) was developed and validated in Brazil^(18)^. The instrument includes 30 items distributed in 6 domains: Practical Activities Execution (D1), Professional communication (D2), Time management (D3), Environment (D4), Professional training (D5), and Theoretical activity (D6). The stress intensity is marked on the questionnaire by the subject according to his/her evaluation to the stress of each situation, being: 0 (I do not experience the situation), 1 (I do not feel stressed with the situation), 2 (I feel a little stressed with the situation) and 3 (I feel very stressed with the situation). To evaluate the result, the corresponding number of stress intensity of the items present in each domain must be summed. The domain with the highest score will be considered predominant and with greater stress intensity. Internal consistency of the domains estimated by Cronbach’s alpha for the original instrument ranged from 0.71 to 0.87^(18)^. In this study, the internal consistency of the domains was analyzed using Cronbach’s alpha coefficient and the following values were obtained: D1 (0.79), D2 (0.81), D3 (0.74), D5 (0.78), D6 (0.68).

The saliva samples were collected by the students themselves at home, using Salivettes, which are plastic tubes with a roll of high-absorption cotton, purchased with funds from the institution where the main researcher worked. Each student was given two Salivettes, identified, individually numbered, and with illustrative pictures (moon and sun) for correct differentiation: night tube (1^st^ collection/moon) and morning tube (2^nd^ collection/sun). Cortisol rhythmicity was evaluated through two saliva samples, at two different times: the first collection was at night, before lying down to sleep (11 pm–12 pm), and the second sample the next day, before getting up, still in bed (6 am–9 am), during the week on school and work days, but not on the week of theoretical and practical tests, or night work, because these circumstances are stimuli that trigger changes in the nocturnal cortisol release rhythmicity, as well as can be triggers for maintaining higher cortisol concentrations, as demonstrated in a previous study^(19)^.

According to the protocol established by the Laboratory of Stress Study (Labeest) of the Institute of Biology (IB) at the Universidade Estadual de Campinas (Unicamp), responsible for the whole packaging process and analysis steps of the saliva samples, the students were instructed not to brush their teeth, not to floss, not to eat, drink or smoke thirty minutes before the collection, not to do any physical activity, and to keep the Salivet in the oral cavity for approximately five minutes or until it was completely soaked with saliva. After collection, the salivettes were kept at room temperature until delivery to the main researcher, who in turn kept them in a refrigerator until they were forwarded to Labeest, where the samples were centrifuged for 20 minutes at 40000 rpm at 4ºC, and the supernatant was frozen at –20ºC until the final assay. In the final assay, samples were analyzed, in duplicate, by the ELISA method, using a commercial DBC kit (DiagnosticsBiochem Canada Inc.Ref CAN-C290^(9)^. Data were expressed in nmol/L for each sample.

### Data Analysis and Processing

The collected data were input into the Excel for Windows (Microsoft Office 2016) and exported to the statistical software SPSS version 23 and GraphPadPrism9 to perform descriptive and inferential analyses. Data distribution was through the Shapiro-Wilk test in situations where the variables or groups being compared presented up to 50 observations and through the Kolmogorov-Smirnov test in situations where the variables or groups being compared presented more than 50 observations. The paired or unpaired Student’s t-test was used for comparisons where the data presented a Normal distribution and the Mann-Whitney test was used for comparisons where the distribution assumption was not met for salivary cortisol data between graduation years and gender. To compare graduation years with salivary cortisol collections, multiple linear regression models were applied via generalized linear models, adjusted for the variables: gender, physical activity, work activity, and alcoholic beverage (confounding variables). These variables were defined as control variables because they have been shown in the literature to be the most commonly used sociodemographic variables for comparative purposes of stress in these populations^(20)^. The comparisons between graduation years and stress domains were through the non-parametric Kruskal-Wallis test, followed by Dunn’s post-test. The significance level adopted was 5%.

### Ethical Aspects

According to Resolution 466/2012 of the National Health Council, ethical aspects were respected, referring to recommendations for research with human beings, and all participants signed an Informed Consent Form (ICF). The study was approved by the Research Ethics Committee with Human Beings of the Campinas State University with protocol no. 1,799,914/2016.

## RESULTS

The descriptive data showed that 187 students answered the questionnaires, 152 women (81.29%), 35 men (18.71%), with a mean age of 26.8 years (SD: 8.03), 126 single (67.38%), 129 without children (68.99%), 154 living in the city (82.36%), and 139 working (74.33%). Regarding lifestyle habits, 123 were not physically active (65.78%), 93 reported drinking alcohol (49.73%), and 177 (94.66%) were nonsmokers.

According to Dunn’s post-test, the domains such as Practical Activities Execution (D1), Professional Communication (D2), and Professional Training (D5) showed higher mean values for the 3^rd^, 4^th^, and 5^th^ years compared to the 1^st^ and 2^nd^ years. Higher mean scores were recorded in the domains Professional Communication (D2) and Professional Training (D5) for the 5^th^ year compared to the 3^rd^ year, and the domain Environment (D4) showed a higher mean for the 5^th^ year compared to the 1^st^ and 3^rd^ year of the course (Table 1).

**Table 1.** Comparison by graduation year with stress domains – Poços de Caldas, MG, Brazil, 2018.

Stress Domains	Year	Students (n)	Mean (SD)	p-value
Practical Activities Execution (D1)**	1	35	3.80 (2.64)	< 0.0001*
	2	30	5.73 (2.32)	
	3	40	9.35 (3.67)	
	4	28	9.43 (4.07)	
	5	54	10.70 (2.92)	
Professional Communication (D2)**	1	35	1.43 (1.74)	< 0.0001*
	2	30	1.57 (1.70)	
	3	40	4.10 (2.91)	
	4	28	4.86 (3.32)	
	5	54	6.57 (2.25)	
**Time Management (D3)**	1	35	8.26 (3.09)	0.0906*
	2	30	8.90 (4.02)	
	3	40	8.38 (3.84)	
	4	28	9.96 (2.52)	
	5	54	9.89 (3.54)	
Environment (D4)**	1	35	2.57 (1.94)	< 0.0001*
	2	30	4.63 (3.11)	
	3	40	3.73 (3.60)	
	4	28	4.46 (2.53)	
	5	54	6.02 (3.31)	
Professional Training (D5)**	1	35	5.83 (1.98)	< 0.0001*
	2	30	6.07 (1.95)	
	3	40	9.43 (4.28)	
	4	28	10.50 (3.44)	
	5	54	11.94 (3.78)	
**Theoretical Activity (D6)**	1	35	9.49 (2.74)	0.6734*
	2	30	10.23 (2.54)	
	3	40	9.33 (3.56)	
	4	28	9.50 (2.69)	
	5	54	9.30 (2.67)	

*p-value obtained using Kruskal-Wallis test; **p-value significant in Dunn’s post-test on comparisons: D1 (1 x 3; 1 x 4; 1 x 5; 2 x 3; 2 x 4; 2 x 5); D2: (1 x 3; 1 x 4; 1 x 5; 2 x 3; 2 x 4; 2 x 5; 3 x 5); D4: (1 x 5; 3 x 5); D5: (1 x 3; 1 x 4; 1 x 5; 2 x 3; 2 x 4; 2 x 5; 3 x 5).

**Figure 1.**
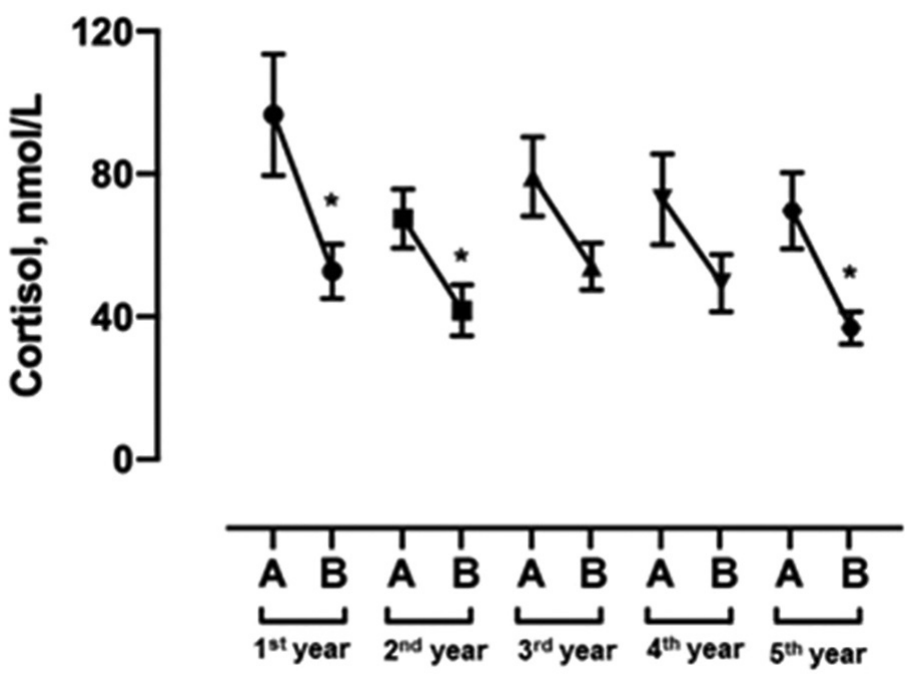
Cortisol rhythmicity by graduation year, Poços de Caldas, MG, 2018. (* = significant p-value; data are presented as the mean and standard error of the mean; saliva samples were collected at two different times. The set points are: on waking (6–9 am) and before bedtime (11–12 pm). Note A: awake; B: before bed.

As for salivary cortisol, 129 students participated. Significant values were obtained between collection times for the 1st year (p = 0.0319) by the Mann-Whitney test, 2nd (p = 0.0245), and 5th (p < 0.0001) years (unpaired t-Student test) (Figure 1).

Table 2 presents the multiple linear regression model between salivary cortisol collections and undergraduate years adjusted for the confounding variables, gender, physical activity, work activity, and alcoholic beverage. There were no significant results.

**Table 2.** Comparison between graduation years and salivary cortisol collections – Poços de Caldas, MG, Brazil, 2018.

	Year	n	Mean (SD)	95%CI Coefficient (L.I L.S)	p-value*
Collection on awakening	1	19	96.58 (74.16)	Reference	Reference
	2	17	67.45 (33.97)	–32.81 (–73.96; 8.35)	0.1182
	3	23	79.26 (52.94)	–13.24 (–50.74; 24.26)	0.4888
	4	24	72.89 (62.05)	–22.62 (–59.52; 14.28)	0.2295
	5	46	69.68 (72.49)	–20.96 (–54.13; 12.21)	0.2156
Collection before Bed time	1	19	52.71 (33.27)	Reference	Reference
	2	17	41.78 (29.28)	–17.09 (–37.77; 3.60)	0.1054
	3	23	54.06 (31.39)	1.96 (–16.88; 20.81)	0.8381
	4	24	49.39 (39.26	–3.11 (–21.66; 15.44)	0.7428
	5	46	36.80 (30.75)	–15.42 (–32.09; 1.25)	0.0699

*p-value obtained through multiple linear regression model adjusted by the variables gender, physical activity, work activity, and alcoholic beverage. C.I.: Confidence Interval. L.I.: Lower Limit; S.L.: Upper Limit.

**Figure 2.**
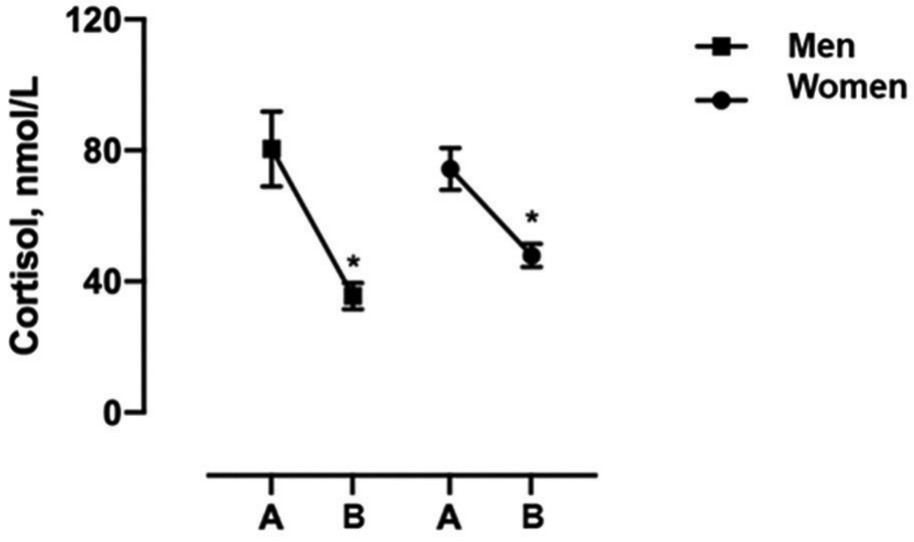
Cortisol collections rhythmicity by gender, Poços de Caldas, MG, 2018. (* = significant p-value; data are presented as the mean and standard deviation of the mean; saliva samples were collected at two different times. The set points are: on waking (6–9 am) and before bedtime (11–24 pm). Note A: awake; B: before Bed time.

When the salivary cortisol collections were compared between the genders using the Man-Whitney test, no significant results were obtained for the collections upon waking (p = 0.7533) and before sleeping (p = 0.1532). The data regarding the times of the two cortisol collections for each sex showed significant values for both men (p < 0.0001) and women (p < 0.0001) (Figure 2).

## DISCUSSION

The psychological stress of undergraduate nursing students has been analyzed in the national scenario^(3)^ and other world regions^(2)^, but from the physiological perspective, national studies are still incipient. Thus, for the analyses regarding cortisol data, other similar populations will be considered.

The profile data and lifestyle habits obtained in this study corroborate with results from research conducted in a private institution in São Paulo state where nursing courses are held at night, however, as for the variable consumption of alcoholic beverages, the result was slightly higher, 52.06%, compared to the findings of this study, 49.73%, and also identified divergent results regarding the residence place since only 41.15% of students lived in the same place as the college^(21)^.

Concerning psychological stress, domain 1 Practical Activities Execution, related to the conduction of procedures, domain 2 Professional Communication, which represents interpersonal relationship difficulties and communicability with professionals in adverse situations that may arise in practice fields or supervised internship, and domain 5 Professional Training, which reflects the concerns and aspirations related to practice and professional training^(21)^, were the greatest stress factors for students in the 3^rd^, 4^th^, and 5^th^ years compared to the initial stages. Also, domain 2 Professional Communication, and domain 5 Professional Training were the greatest stressors for students in the 5^th^ year compared to the 3^rd^ year.

On these data, we consider that there is a gradual exposure to these factors as the course progresses, and in the final phase students are more exposed to pre-professional work, in addition to insecurity and concerns to enter the labor market, and prospects as a professional nurse^(22)^, which requires greater skills and competence for more effective communication with different health professionals, similar challenges were identified in comparative research between nursing and medical students and proposes an improvement in divergent communicability between teachers and supervisors^(23)^.

Domain 4, Environment, represented an even greater stress factor for final-year students compared to first and third-year students. These findings indicate the difficulties in commuting between home and the college or internship fields, or even the adverse situations with public transportation. The last year is the mandatory internship, which corresponds to a higher performance of care practices and there is greater displacement in the different internship sites^(24)^.

Given the evidence on psychological stress among students, research shows that regular physical activity, intervention measures such as meditation, and resilience skills training have been linked to improvements in overall well-being, stress reduction, and better preparedness to prevent burnout and stress-related illness^(25)^.

We observed that there was no statistical relevance in salivary cortisol concentrations and graduation years, however, a significant reduction between cortisol collection times was evidenced for 1st, 2nd, and 5th-year students, thus demonstrating preserved cortisol rhythmicity when compared to 3rd and 4th-year students. Consider that although in the initial stage the mean cortisol values in the morning were high, which may reflect a possible acute stress response that may occur when behavioral or cognitive responses are not yet well developed, or when the challenging situation is intense, new, or unexpected^(26)^, there was a significant reduction among collection times in this stage. The second year recorded lower cortisol values in the morning, suggesting a possibly more adaptive response to the stressors of the initial stage, and also a significant reduction in collection times^(27)^.

In contrast, 3^rd^ and 4^th^-year students showed little reduction in values between cortisol collections, especially 3rd years, who had the highest mean cortisol values before bedtime. Therefore, this suggests that at these stages there may have been a greater allostatic overload, less ability to cope with potentially stressful situations, and less adaptive reactions and possible disruption of the HPA. The increase in evening cortisol over time may be linked to fatigue, as suggested by other investigations^(28)^. In the last year, however, evening cortisol was lower with a significant result between cortisol collection times, which may indicate a return to the standard HPA axis functioning^(9)^.

The data also indicated no possible interference of confounding variables adjusted in the multiple linear regression model in cortisol collections, showing a homogeneous population, and agree with the Brazilian research results of biology undergraduates, in which cortisol rhythmicity was preserved even under psychosocial stress^(29)^. Regarding the gender variable, there was a significant reduction between cortisol collection times, but not between genders. Cortisol concentration may be different between genders^(19)^, since men and women may have different reactions to psychological^(3)^ and physiological^(19)^ stress. Additionally, higher variability in cortisol measurement in women is assumed due to hormonal fluctuation, caused by factors such as menstrual cycle and use of oral contraceptives^(30)^, however, these were not analyzed in this study, but the data obtained corroborate with other studies^(9)^.

This study is limited since it was carried out in a private higher education institution, with students from the evening course, and therefore cannot be generalized. Thus, studies should be prospectively analyzed in public institutions, and courses offered during daytime and full-time periods. Determining the number of salivary cortisol samples may be another limitation, and we suggest that future studies should include more consecutive days and times for a better analysis of the cortisol circadian rhythm.

This study showed stress factors prevalence and salivary cortisol values in each graduation stage, which can help in the awareness of managers and students in building prevention and promotion measures for mental health and better quality of academic activities in each stage of the course according to the institutional reality, aiming at the earlier identification of academic stressors, as well as possible interventions for the reduction of stress levels and possible changes in physical, psychological and professional health of future nurses.

## CONCLUSIONS

The study found about psychological stress that students from the 3^rd^ to 5^th^ year had higher mean values for stress factors related to the domains of Practical Activities Execution, Professional Communication, and Professional Training compared to the initial stages. For the final year students, it was the Professional Communication and Professional Training domains compared to the 3^rd^ year, and the Environment domain compared to the 1st and 3rd years of the course. There was no difference in cortisol concentrations between genders and undergraduate years, but a significant difference was found between cortisol collection times for males, females, and students in the 1^st^, 2^nd^, and 5^th^ years of the course, with better cortisol production adjustment in these undergraduate stages.
